# Invasive pneumococcal diseases in children and adults before and after introduction of the 10-valent pneumococcal conjugate vaccine into the Austrian national immunization program

**DOI:** 10.1371/journal.pone.0210081

**Published:** 2019-01-10

**Authors:** Lukas Richter, Daniela Schmid, Elisabeth Eva Kanitz, Ines Zwazl, Eva Pöllabauer, Joanna Jasinska, Heinz Burgmann, Michael Kundi, Ursula Wiedermann

**Affiliations:** 1 Department of Infectious Disease Epidemiology, Austrian Agency for Health and Food Safety, Vienna, Austria; 2 Institute of Specific Prophylaxis and Tropical Medicine, Center for Pathophysiology, Infectiology and Immunology, Medical University of Vienna, Vienna, Austria; 3 General Hospital, AKH, Vienna, Austria; 4 Institute of Environmental Health, Center for Public Health, Medical University of Vienna, Vienna, Austria; Universidade de Lisboa Faculdade de Medicina, PORTUGAL

## Abstract

**Background:**

In February 2012 the ten-valent pneumococcal conjugate vaccine (PCV10) with a 2+1 doses schedule (3, 5, 12 or 14 months of age) without catch-up vaccination was introduced in Austria. We assessed direct and indirect vaccine effects on invasive pneumococcal disease (IPD) by a population-based intervention study.

**Methods:**

The study period was divided into pre- (2009–2011) and post-period (2013–2017, February), regarding 2012 as transition year. Outcomes were defined as PCV10 ST-IPD, the PCV10-related ST 6A and 19A IPD and non-PCV10 excluding ST 6A-/19A-IPD (NVT-IPD). We used national surveillance data and compared average monthly incidence rate (IR) between pre- and post-period among <5, 5–49 and ≥50 years old. Additionally, for the 5–49 and ≥50 years old, and the 50–59 and ≥60 years old, we analyzed monthly incidence data of the pre-, post-period, and estimated trend and level changes by using a segmented time-series regression.

**Results:**

The PCV-10 IPD was reduced by 58% (95% CI: 30%; 74%) and 67% (95% CI: 32%; 84%) among <5 and ≥50 years old; the reduction in ≥60 years was 71% (95% CI: 36%; 88%). There were no significant changes in the pre-post-rate or incidence trend of NVT-IPD in the <5 and ≥50 years old. ST-specific analyses revealed no ST 6A- and ST 19A IPD decline in any age-group, and a ST 8 IPD increase among ≥50 years old (IR ratio: 3.5; 95% CI: 1.7; 7.2). We found no vaccine effects among 5–49 years old.

**Conclusions:**

Our study adds to the evidence on direct and indirect protection of a childhood PCV10 vaccine program. Elderlies seem to benefit the most. Findings did not support PCV 10 cross-protection, but indicate replacement at least for ST 8 among the ≥50 years old. Follow-up analyses of IPD surveillance data are needed to fully characterize the magnitude of serotype replacement and further vaccine-attributable IPD reduction with time.

## Introduction

*Streptococcus pneumoniae* (pneumococcus), a gram-positive diplococcus, is adapted to colonize the human nasopharynx (NP). Pneumococcal NP colonization is prevalent among children during the first few years of life and declines with age. Depending on host and agent factors, pneumococcus may spread in the respiratory tract, causing non-invasive pneumococcal diseases or through the bloodstream to other sites, leading to invasive pneumococcal diseases (IPD) such as meningitis, septicemia, bacteremic pneumonia, osteomyelitis or arthritis [1]. The highest burden of serious pneumococcal disease occurs in infants, the <2 years old, and the elderly [2–4]. Prior to the widespread introduction of pneumococcal conjugate vaccines (PCV) into national childhood immunization programs (NIPs), Europe and the United States reported annual rates of IPD between 11 and 70 per 100,000 persons [1,5–7].

Conventional serotyping and molecular analyses have identified at least 97 different pneumococci capsular polysaccharide STs [8]. Two types of pneumococcal vaccines, i.e. unconjugated and conjugated, are currently available for use in humans, varying with respect to design, composition, target populations, immunogenicity and efficacy. The unconjugated pneumococcal polysaccharide vaccine is a 23-valent vaccine (PPV23), and the pneumococcal polysaccharide conjugate vaccines (PCV) are available in several formulations. The first pneumococcal polysaccharide conjugate vaccines (PCV), a 7-valent PCV (PCV7) covering the STs 4, 6B, 9V, 14, 18C, 19F, 23F, became available in 2001 and was introduced into national immunization programs (NIPs) of many European countries. In 2009, PCV10 (covering PCV7 STs plus STs 1, 5, 7F) and PCV13 (covering PCV10 STs plus STs 3, 6A, 19A) became available, replacing PCV7 in most European countries. All PCVs are characterized by markedly improved immunogenicity in neonates and young children [9]. Unlike PPV23, the PCVs prevent NP colonization of vaccine serotypes, thereby conferring protection in unvaccinated and vaccination-ineligible individuals as a result of diminished transmission of vaccine serotypes (indirect vaccine effect) [10,11]. The impact of PCV introduction into NIPs has exceeded the IPD reducing effects observed in vaccine efficacy studies due to the indirect PCV effects [12–14]. The magnitude of indirect vaccine effects depends on age of the non-vaccine target population, PCV coverage of the vaccine-target population and the time elapsed since nationwide PCV implementation [15]. Following the routine use of PCV7 in many countries worldwide for over ten years, a systematic review of vaccine effectiveness studies found a median before-after rate reduction in VT-IPD among all age-groups of 65.5% [6]. After switching from PCV7 to PCV13, many European countries, such as the United Kingdom, Denmark, Spain, France and Norway, and the US reported a pronounced decrease in IPDs due to STs additionally covered by PCV13 (defined as PCV13-PCV7 ST-IPD) among vaccinated and also non-vaccinated population groups [16–21].

More than 30 countries worldwide have introduced PCV10 in their NIPs, of which several demonstrated high vaccine efficacy and effectiveness among vaccinated and unvaccinated children and adults such as Finland, Kenya and Chile (PCV7-naïve countries), and New Zealand and the Netherlands (with PCV7 before PCV10 introduction) [22–28].

A vaccination-induced increase in non-vaccine type (NVT) colonization, referred to as "replacement" carriage, may potentially lead to increased NVT disease rates in both vaccinated and unvaccinated populations. Findings on the extent of NVT replacement in carriage and disease following introduction of PCVs vary, depending on factors such as previous vaccine and antibiotics use, vaccination program (VP) details, vaccination coverage, surveillance activity and, in particular, on the methods used for trend analyses to quantify the vaccination contribution [29]. NVT replacement disease has been reported in the era of PCV7 and after introduction of PCV13 and PCV10. However, in young children the rise in NVT-IPDs is generally smaller than the decrease in VT-IPDs, resulting in a net benefit. For older adults, some countries (Norway, The Netherlands, US, Denmark) reported decreases in overall IPD, while others (Finland, New Zealand, Quebec, France, South Africa, Sweden, Germany, UK) did not [17,18,20,24,30–37].

In Austria, based on national sales figures (MAT data) as per pharmacy statistics, the estimated uptake of PPV23, which has been recommended, but not publically funded, since 1998 for ≥50 years old, was 5.7 doses per 1000 adults of ≥50 years between July 2017 and July 2018 (the only available data), assuming all doses administered in this age group. A dual recommendation of PPV23/PPV13 for all ≥50 years old and high-risk groups was introduced in 2013. The PCV13 uptake was estimated at 9.2, 7.3 and 9.1 doses per 1000 adults of ≥50 years for the years 2016, 2017, 2018.

Similar to Finland and The Netherlands, having introduced a PCV10 (2+1 doses) VP in 2010 and 2011, the Austrian Ministry of Health introduced the PCV10 vaccination with a 2+1 doses schedule (3, 5, and 12 or 14 months of age) without catch-up vaccinations into the publically funded childhood NIP in January 2012. All children born January 1st, 2012 or thereafter were eligible for vaccination.

The aim of our study was to evaluate for the first time the effects of the PCV10 (2+1 doses) VP, on vaccine-type (VT) IPD and non-VT IPD in children and adults in Austria.

## Methods

### Data-sources and study cases

In 2002, IPD became notifiable in Austria. Since this year, active surveillance of IPD among <5 years old patients of all pediatric wards in Austrian hospitals has been operated by the Institute of Specific Prophylaxis and Tropical Medicine (ISPTM) at the Medical University Vienna and supported by third party funding [38]. The data were collected after signed informed consent, and a report form including the clinical cases, demographic parameters, such as age and gender along with the initials were sent from the pediatric units to the Institute of Specific Prophylaxis and Tropical Medicine at the Medical University Vienna. The collected data sets were matched with those of AGES to complete the number of IPD cases. This IIR of prospective active surveillance of IPD in infant < 5 years was approved by the Ethics Committee of the Medical University Vienna (013/06/201/, EKnr 252/2008).

In 2009, the national surveillance system for all mandatorily notifiable infectious diseases was improved in acceptability and simplicity by introducing a nation-wide, web-based-case recording system to which clinicians and laboratories report cases according to the EU case definitions for surveillance [39]. Since then, the Austrian Agency for Health and Food Safety (AGES) has been responsible for data quality assurance and regular analyses.

We used the national surveillance data from January 2009 until February 2017 on IPD, including information on age, clinical presentation, and month of diagnosis and on serotype for each case of IPD, complemented with data from the national reference laboratory for pneumococci at AGES. Cases identified through active surveillance of the Austrian pediatric wards were cross-checked with cases reported through the national surveillance system and case data added if necessary. The annual population data were obtained from Statistics Austria.

A case of IPD was defined according to the laboratory-based EU surveillance case definition 2012 [39], as a patient with a specimen obtained from a normally sterile site (e.g. blood, cerebrospinal fluid), which tested culture or PCR positive for *S*. *pneumoniae*. Isolates were serotyped by Quellung reaction at the National reference laboratory for pneumococci, as described elsewhere [40].

We estimated the vaccination coverage for ≥2 PCV10 doses among children in the 2^nd^ year of life for the birth cohorts 2009–2015, and among the <5 years old for the years 2009 until 2016 through dividing the birth cohort specific number of ≥2 doses administration by the respective birth cohort. We used data from the provincial vaccine registers of the nine Austrian provinces, and data on the annual birth cohorts provided by the provincial public health authorities and Statistics Austria.

### Study design, intervention and outcomes

For the population-based intervention study, we used a non-experimental classical before-after study design with pre-post comparison of average rates for the age groups <5, 5–49, and ≥50 years old, and the age-subgroups <2, 2–4, 50–59 and ≥60 years. Furthermore, we applied an interrupted time series (ITS) design with segmented regression for the 5–49 and ≥50 years old, and the age-subgroups 50–59 and ≥60 years. The intervention under study was the introduction of a PCV10 (2+1 doses) VP in the publically funded NIP in January, 2012 without catch-up vaccinations. The study period February 2009 until February 2017 was divided into the pre-intervention period (i.e. pre-period), from January 2009 to December 2011 and into the post-intervention (post-period), from January 2013 until February 2017. The year 2012 was excluded from the study as the transition period.

Outcomes were defined as the group of IPDs due to PCV10 serotypes (PCV10 ST-IPD; ‘intervention outcome’) and IPDs due to the serotypes 6A and 19A, for which PCV10 cross-protection is discussed (ST 6A IPD, ST 19A IPD; ‘intervention-related outcome’). In order to assess a PCV10-VP induced increase of non-vaccine serotype IPDs (‘replacement outcomes’): ST 3 IPD, and the group of IPDs due to serotypes other than PCV10 STs, while excluding cases of ST 6A and ST 19A IPD (referred to as non-PCV10 ex ST 6A-/19A-IPD).

Outcomes for assessing net changes were serotyped IPD (i.e. PCV10 ST-IPD and non-PCV10 ST-IPD) and overall IPD, comprising non-serotyped and serotyped IPD. Table 1 lists the age-group-specific outcomes under study by category (intervention, replacement, net change) including their definition, and specifies the methodologies (pre-post rate comparison, segmented regression analysis) applied for measuring the outcome changes.

**Table 1:** Age-group specific outcomes by category, their definition and the applied methodology (pre-post rate comparison indicated by A, segmented regression analysis of ITS by B).

Age-group in yrs	Outcome category	Outcome definition	Methodology
**<5**	intervention	PCV10 ST-IPD	A
	intervention-related	ST 6A IPD	A
	intervention-related	ST 19A IPD	A
	replacement	non-PCV10 ex ST 6A-/19A-IPD	A
	replacement	ST 3 IPD	A
	net change I	serotyped IPD	A
	net change II	overall IPD	A
<2	intervention	PCV10 ST-IPD	A
	replacement	non-PCV10 ex ST 6A-/19A-IPD	A
	net change I	serotyped IPD	A
	net change II	overall IPD	A
2–4	intervention	PCV10 ST-IPD	A
	replacement	non-PCV10 ex ST 6A-/19A-IPD	A
	net change I	serotyped IPD	A
	net change II	overall IPD	A
**5–49**	intervention	PCV10 ST-IPD	A and B
	intervention-related	ST 6A IPD	A
	intervention-related	ST 19A IPD	A
	replacement	non-PCV10 ex ST 6A-/19A-IPD	A and B
	replacement	ST 3 IPD	A
	net change I	serotyped IPD	A and B
	net change II	overall IPD	A and B
**≥50**	intervention	PCV10 ST-IPD	A and B
	intervention-related	ST 6A IPD	A
	intervention-related	ST 19A IPD	A
	replacement	non-PCV10 ex ST 6A-/19A-IPD	A and B
	replacement	ST 3 IPD	A and B
	net change I	serotyped IPD	A and B
	net change II	overall IPD	A and B
50–59	intervention	PCV10 ST-IPD	A and B
	replacement	non-PCV10 ex ST 6A-/19A-IPD	A and B
	net change I	serotyped IPD	A and B
	net change II	overall IPD	A and B
≥60	intervention	PCV10 ST-IPD	A and B
	replacement	non-PCV10 ex ST 6A-/19A-IPD	A and B
	net change I	serotyped IPD	A and B
	net change II	overall IPD	A and B

### Descriptive analyses

All IPD cases were described by their clinical presentation and availability of serotype information. We calculated the proportion of PCV10 STs among the serotyped IPD cases of the pre-period for the <5, 5–59 and ≥50 years old.

### Statistical analyses

We compared the <5, 5–49 and ≥50 years old, in addition the <2, 2–4, 5–49, 50–59, ≥60 years old, of the post-period, defined as the intervention population (i.e. exposed to the VP including vaccinated and unvaccinated) to the corresponding age-groups of the pre-period (reference population) with respect to the period-specific monthly average incidence rate (IR) per 100,000 person-months (pm). The period-specific IRs were calculated through dividing the period-specific number of IPD cases by the period-specific mid-term population multiplied by the respective number of observation months (pre-period: 36 months; post-period: 50 months). The vaccine effects were measured through calculating the IR difference per 100,000 pm between the pre- and post-period (pre-post IR difference: *IR_pre_* − *IR_post_*) and the IR ratio (IRR: $\frac{IR_{post}}{IR_{pre}}$). A Chi^2^ test was used to test for significance of the pre-post IR difference and IR ratio. We calculated for the <5 years old, and the age-subgroups <2 and 2–4 years, the proportionate reduction in post-period IR of the intervention and net change outcomes relative to the pre-period (vaccine effectiveness, VE), using the formula $VE(\%)=1-(\frac{IR_{post}}{IR_{pre}})\;x\;100$. The corresponding 95% confidence intervals (95% CI) was calculated by using the method described by Wald [41]. Among the <5 years old, for identifying pre-, post-intervention time trends in the outcomes of interest, we additionally calculated average percent change (APC) in the monthly incidence for the pre-period and post-period using two separate negative binomial regression models.

For age groups 5–49 years and ≥50 years old, and the age-subgroups 50–59 and ≥60 years, we additionally applied the interrupted time series regression. We used a segmented negative binomial regression model, including a seasonal component, on the monthly IPD incidence of the period January 2009—February 2017 in order to compare time series data on IPD of the pre-period with that of the post-period [42,43]. The model can be written as:

$$log(Y_{t})=\log\left(pop_{t}\right)+\beta_{0}+\beta_{1}t+\beta_{2}sin\left(\frac{2\pi t}{12}\right){+\beta }_{3}cos\left(\frac{2\pi t}{12}\right)+\beta_{5}{\left(t-t_{o}\right)}^{+}+{\delta (t-t_{o})[\beta_{4}+\beta }_{6}\sin\left(\frac{2\pi t}{12}\right){+\beta }_{7}\cos(\frac{2\pi t}{12})]+e_{t}.$$

Here, Y_t_ is the number of IPD cases observed in month t; pop_t_ is the population size in 100,000 at month t, t_0_ is the last month (month 48) of the pre-period including the transition time, *δ*(*x*) is the indicator function (it is 0 if x≤0 and 1 if x>0), (*x*)^+^ is the cut off operator (it is x if x>0 and 0 otherwise), seasonality is modelled as a sinusoidal function (sin and cos terms to account for a phase shift), and e_t_ denotes the residual. The parameters of the model can be interpreted as follows: β_0_ estimates the number of cases per 100.000 in the month at the beginning of the pre- period (i.e. baseline level), β_1_ estimates the linear trend, and β_2_ and β_3_ the seasonality of the monthly incidence of the pre-period (i.e. baseline trend and seasonality); β_4_ estimates the change (jump or drop) in the level of the incidence at the post-period beginning, and β_5_, β_6_ and β_7_ the change in trend (slope increase or decrease) and seasonality of the monthly incidence during the post-period, compared to the pre-period (i.e. baseline). The vaccine effects were expressed by percent change in post-period trend and post-period level, relative to the pre-period trend and level of the monthly incidence (% change/month, % change), calculated through inserting β_4_ and β_5_ in the formula: *f*(*x*) = (exp(*x*) − 1) * 100. The 95% confidence intervals (95% CI) were calculated using the bootstrap method by resampling model residuals 5000 times [44–46]. As a second vaccine effect measure, we calculated, based on the above explained model, the proportionate reduction in the post-period IPD, relative to the pre-period (i.e. VE) by using the formula $VE\;(\%)=\frac{n_{noVP}-n_{VP}}{n_{noVP}}\;x\;100$. Here, n_noVP_ refers to the expected number of cases in the population of the post-period, if there was no VP, and n_VP_ refers to the modelled number of IPD cases based on the observed number of cases in the population of the post-period (i.e. population exposed to the VP). The 95% CI was calculated using the bootstrap method, as described above.

To describe pre-post changes in the occurrence of the serotype-specific IPDs, we calculated annual average incidence rates (IR) per 10^6^ pm of ST-specific IPD in the <5 years old and ≥50 years old. We ranked the ten most frequent IPD causing serotypes of the pre-period (2009–2011), the early post-period (2013/2014) and late post-period (2015–2016) from rank 1 (R1) to R10 by the IR per 10^6^ pm or alphanumerically, in case of equal IR. The ST-specific IPD IR ratios (IRR) for the ten most frequent STs, comparing early and late post-period with the pre-period were tested for significance using Chi^2^ tests. In order to identify pre-, post-intervention time trends in the monthly incidence of ST-specific IPDs, we additionally estimated the monthly APC for the pre- and post-period by two separate negative binomial regression models, when a segmented negative binomial regression model was not appropriate due to too small cases numbers.

Additionally, we illustrated among the <5 and ≥50 years old the top 20 and 25 IPD causing serotypes by the annual average IR per 10^6^ pm using a scatterplot based on frequency distribution rank for the pre-period and late post-period. All statistical analyses were performed using the R-software version 3.4.1 [47]. We considered a p-value of <0.05 to be significant.

## Results

### Study cases and descriptive analysis

During the study period 2009–2017, 2952 cases of IPD were identified in the Austrian population, 2905 cases through the national surveillance system and additional 47 cases through active surveillance at the Austrian pediatric wards (including 13 cases in 2009 and 6, 7, 8, 5, 4, 1, 3 and 0 cases in the years 2010-02/2017). Of the 2952 IPD cases, information on clinical presentation was available for 2923 cases (99%). Bacteremic pneumonia was the most frequent clinical presentation (1404; 48%) followed by primary bacteremia (1063; 36%), meningitis (430; 15%) and empyema with bacteremia or pneumonia (26; 1%). Of the 2952 identified and recorded cases, in 2635 (89%) information on ST was available and these cases were included in the analyses of serotyped IPD cases; 172 (73%) serotyped cases of a total of 237 were in <5 years old, 447 (88%) serotyped cases of 507 were in 5–49 years old and 2016 (91%) serotyped cases of a total of 2208 were in ≥50 years old adults. The proportion of cases serotyped among the <5 and 5–49 years old did not differ significantly between the pre- and post-period (<5 years old: 71.3% (pre), 73.3% (post); 5–49 years old: 88.6% (pre), 88.4% (post)). Among the ≥50 years old, the post-period proportion of serotyped cases was significantly lower (89.5%) than that of the pre-period (94.2%) with a difference of 4.7% (95% CI: 2.2%; 7.2%).

In the pre-period (2009–2011), the PCV10 STs accounted for 53.2% of the 77 serotyped IPD cases among the <5 years old, with a PCV10 STs proportion of 58.8% of the 51 serotyped IPD cases among the <2 years old, and 42.3% of the 26 serotyped cases among the 2–4 years old. Among the 5–49 years old, 53.8% of the 171 serotyped IPDs and 36.8% of the 638 serotyped IPD cases among the ≥50 years old were PCV10 STs, from which 34.2% out of 146 serotyped cases were among the 50–59 years old and 37.6% of 492 among the ≥60 years old.

**Fig 1:**
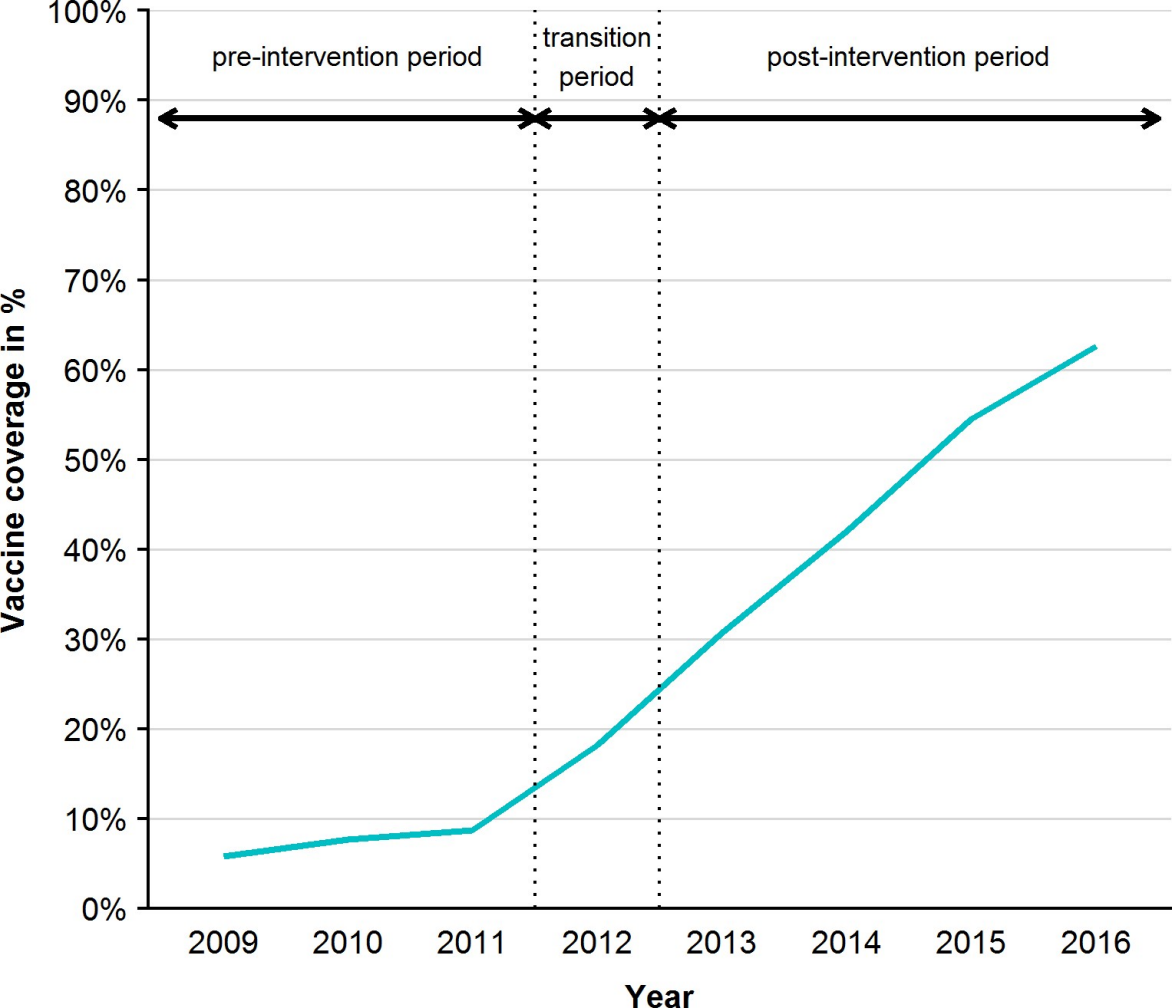
Vaccination coverage for ≥2 doses of PCV10 among the <5 years old per year during the pre-intervention period, transition period and post-intervention period, 2009–2016.

The vaccination coverage for ≥2 doses of PCV10 among infants in the 2^nd^ year of life was 9% for the birth cohort 2009, 10% for the birth cohort 2010, and 30%, 70%, 68%, 69% and 72% for the birth cohorts 2011–2015. Among the <5 years old the cumulative vaccination coverage was 5.8%, 7.7% and 8.7% in the years 2009–2011 (pre-period) and increased gradually from 18.2% in the year 2012 (transition period) to 30.7%, 42.0%, 54.5% and 62.6% in the years 2013–2016 (post-period) (Fig 1).

### Effects of PCV10 in children

#### Effect on PCV10 ST-IPD

Among the <5 years old, the monthly average IR of PCV10 ST-IPD declined from 0.29 in the pre-period to 0.12 per 100,000 pm in the post-period, resulting in a VE of 58% (95% CI: 30%; 74%). The VE was at 77% (95% CI: 53%; 89%) in the <2 years old (Table 2 and S1 Table). Among the 2–4 years old no significant pre-post rate change in the PCV10 ST-IPD was found (Table 2), whereas the pre-period monthly APC, already, was −5.3% (95% CI: −10.9%; −0.1%; incidence level: 0.28/100,000); the post-period monthly APC was −2.8% (95% CI: −6.3%; +0.6%; incidence level: 0.89/100,000).

**Table 2:** Monthly average incidence rates of the pre-period (IR pre) and post-period (IR post), pre-post IR ratios (IRR) and pre-post IR difference (IRD)/100,000 pm with 95% confidence interval (CI) for intervention, replacement and net change outcomes among <5, 5–49 and ≥50 years old, additionally among the age-subgroups <2, 2–4, 50–59 and ≥60 years, Austria, January, 2009-February, 2017.

Age-group	Outcome category	IPD by ST	IRpre	IRpost	IRR (95% CI)	IRD (95% CI)
**<5**	intervention	PCV10 ST-IPD	0.29	0.12	0.42 (0.26; 0.70)	−0.17 (−0.27; −0.07)
	intervention-related	6A IPD	0.02	0.01	0.46 (0.08; 2.78)	−0.01 (−0.04; +0.02)
	intervention-related	19A IPD	0.04	0.07	1.63 (0.62; 4.23)	+0.03 (−0.02; +0.08)
	replacement	non-PCV10 ex ST 6A-/19A-IPD	0.19	0.16	0.85 (0.51; 1.42)	−0.03 (−0.12; +0.06)
	replacement	3 IPD	0.06	0.04	0.62 (0.24; 1.61)	−0.02 (−0.07; +0.03)
	net change I	serotyped IPD	0.54	0.36	0.65 (0.50; 0.85)	−0.27 (−0.44; −0.09)
	net change II	overall IPD	0.76	0.50	0.67 (0.49; 0.92)	−0.18 (−0.33; −0.03)
<2	intervention	PCV10 ST-IPD	0.54	0.12	0.23 (0.11; 0.47)	−0.42 (−0.62; −0.21)
	replacement	non-PCV10 ex ST 6A-/19A-IPD	0.27	0.24	0.87 (0.44; 1.72)	−0.03 (−0.21; +0.14)
	net change I	serotyped IPD	0.92	0.43	0.47 (0.31; 0.73)	−0.48 (−0.77; −0.19)
	net change II	overall IPD	1.19	0.62	0.52 (0.36; 0.75)	−0.57 (−0.90; −0.23)
2–4	intervention	PCV10 ST-IPD	0.13	0.12	0.96 (0.44; 2.08)	−0.01 (−0.10; +0.09)
	replacement	non-PCV10 ex ST 6A-/19A-IPD	0.14	0.12	0.82 (0.38; 1.77)	−0.03 (−0.12; +0.07)
	net change I	serotyped IPD	0.30	0.32	1.05 (0.64; 1.73)	+0.02 (−0.14; +0.17)
	net change II	overall IPD	0.49	0.42	0.85 (0.57; 1.28)	−0.07 (−0.26; +0.11)
**5–49**	intervention	PCV10 ST-IPD	0.05	0.03	0.66 (0.49; 0.89)	−0.02 (−0.03; +0.00)
	intervention-related	6A IPD	0.002	0.002	1.23 (0.29; 5.13)	+0.00 (−0.00; +0.00)
	intervention-related	19A IPD	0.004	0.005	1.37 (0.55; 3.42)	+0.00 (−0.00; +0.01)
	replacement	non-PCV10 ex ST 6A-/19A-IPD	0.04	0.06	1.44 (1.08; 1.92)	+0.02 (−0.00; +0.03)
	replacement	3 IPD	0.01	0.01	0.86 (0.50; 1.48)	0.00 (−0.01; +0.00)
	net change I	serotyped IPD	0.10	0.10	1.02 (0.83; 1.24)	0.00 (−0.02; +0.02)
	net change II	overall IPD	0.11	0.11	1.02 (0.85; 1.22)	0.00 (−0.02; +0.02)
**≥50**	intervention	PCV10 ST-IPD	0.22	0.14	0.62 (0.52; 0.75)	−0.08 (−0.12; −0.05)
	intervention-related	6A IPD	0.03	0.02	0.58 (0.34; 0.99)	−0.01 (−0.02; +0.00)
	intervention-related	19A IPD	0.03	0.05	1.55 (1.05; 2.27)	+0.02 (+0.00; +0.03)
	replacement	non-PCV10 ex ST 6A-/19A-IPD	0.31	0.50	1.62 (1.43; 1.84)	+0.19 (+0.15; +0.24)
	replacement	3 IPD	0.11	0.19	1.73 (1.40; 2.14)	+0.08 (+0.05; +0.11)
	net change I	serotyped IPD	0.59	0.71	1.20 (1.09; 1.32)	+0.12 (+0.06; +0.18)
	net change II	overall IPD	0.63	0.79	1.26 (1.15; 1.39)	+0.17 (+0.10; +0.23)
50–59	intervention	PCV10 ST-IPD	0.13	0.07	0.51 (0.34; 0.77)	−0.06 (−0.10; −0.02)
	replacement	non-PCV10 ex ST 6A-/19A-IPD	0.21	0.22	1.03 (0.78; 1.35)	+0.01 (−0.05; +0.06)
	net change I	serotyped IPD	0.38	0.32	0.85 (0.69; 1.05)	−0.06 (−0.13; +0.02)
	net change II	overall IPD	0.41	0.37	0.92 (0.75; 1.12)	−0.03 (−0.11; +0.05)
≥60	intervention	PCV10 ST-IPD	0.27	0.18	0.67 (0.54; 0.82)	−0.09 (−0.14; −0.04)
	replacement	non-PCV10 ex ST 6A-/19A-IPD	0.37	0.68	1.86 (1.61; 2.15)	+0.32 (+0.25; +0.38)
	net change I	serotyped IPD	0.71	0.95	1.34 (1.20; 1.49)	+0.24 (+0.15; +0.33)
	net change II	overall IPD	0.75	1.05	1.40 (1.26; 1.56)	+0.30 (+0.21; +0.39)

For the <2, 2–4 years old the pre-post rate comparison for ST 3, 6A and 19A IPD were not performed due to the small case numbers

#### Effect on PCV10-related ST-IPDs

The ST 6A IPD pre-post IRD was non-significant (−0.01/100,000 pm). The same holds for the ST 19A IPD pre-post IRD (+0.03/100.000 pm), as given in Table 2. However, the post-period monthly APC in the ST 19A IPD incidence was of +3.4% (95% CI: −0.3%; +7.7%; incidence level: 0.003/100,000) and the pre-period monthly APC of +0.5% (95% CI: −7.3%; +9.0%, incidence level: 0.021/100,000).

#### Effect on non-PCV10 ex ST 6A-/19A-IPD and ST 3 IPD

A non-significant pre-post drop occurred in the non-PCV10 ex ST 6A-/19A-IPD IR and ST 3 IPD IR (Table 2). For the non-PCV10 ST ex 6A-/19A-IPD, a pre-period monthly APC of −0.7% (95% CI: −4.4%; +3.0%, incidence level: 0.158/100,000) and post-period monthly APC of +1.7% (95% CI: −0.6%; +4.0%, incidence level: 0.036/100,000) indicated a trend reversal. Similar was found for the ST 3 IPD, which showed a pre-period monthly APC of −1.3% (95% CI: −7.8%; +5.3%; incidence level: 0.06/100,000) and post-period monthly APC of +2.6% (95% CI: −1.9%; +8.0%; incidence level: 0.004/100,000).

#### Net change

The monthly average IR of overall IPD among the <5 years old decreased from 0.76 in the pre-period to 0.50 per 100,000 pm in the post-period, resulting in a proportionate rate reduction of 35% (95% CI: 15%; 50%). The proportionate pre-post rate reduction in serotyped IPD was 33% (95% CI: 8%; 51%) (S1 Table). A greater net benefit was found in the <2 years with a proportionate rate reduction of 48% (95% CI: 25%; 64%) in overall IPD and of 53% (95% CI: 27%; 69%) in serotyped IPD (S1 Table). No net changes were found in the 2–4 years old (Table 2).

### Effects of PCV10 in 5–49 years old

**Fig 2:**
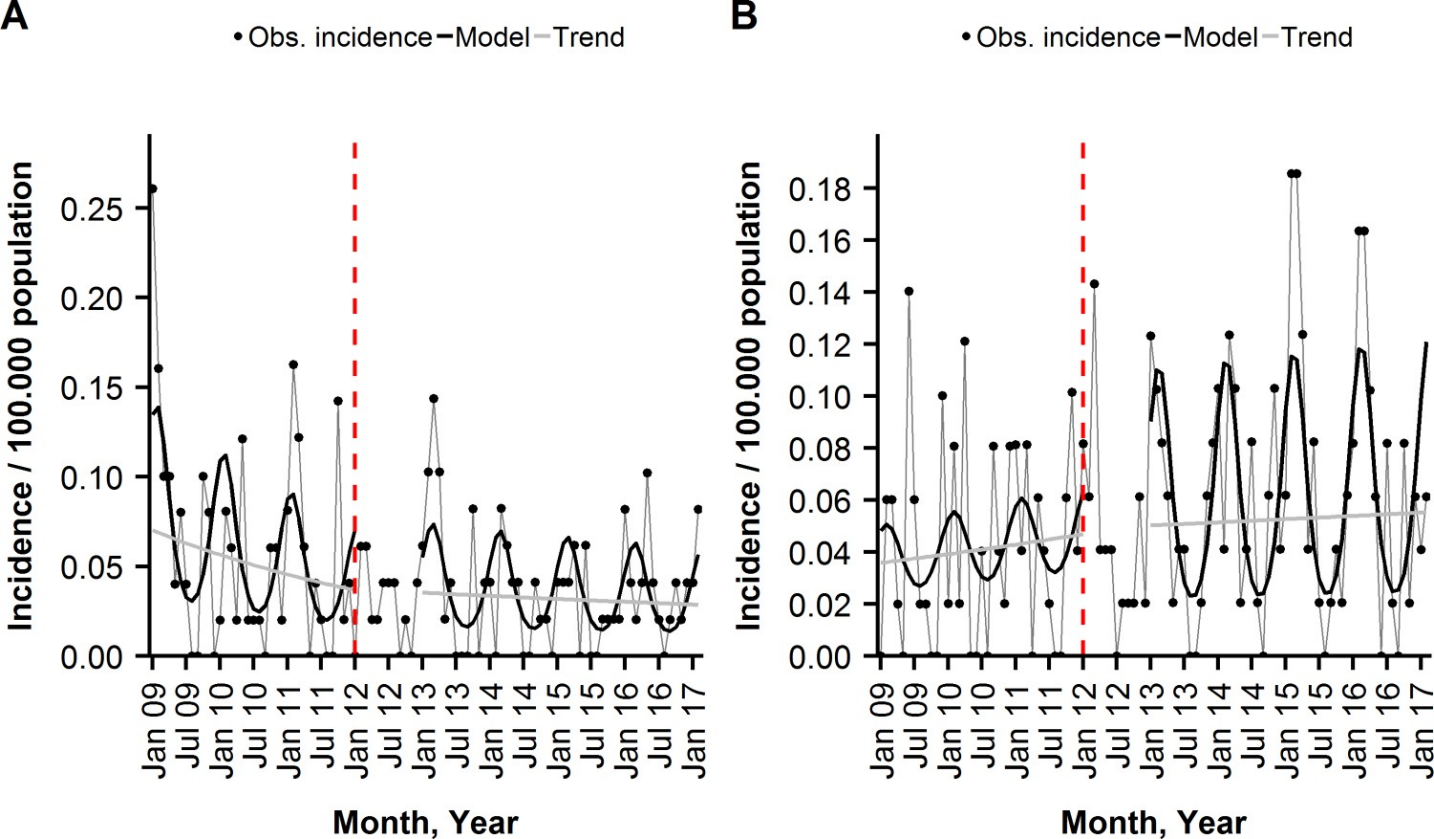
**Monthly incidence of (A) PCV10 ST-IPD, and (B) non-PCV10 ST ex ST 6A-/19A-IPD per 100,000 population among the 5–49 years old, observed and modelled by a segmented negative binominal regression, Austria, January, 2009-February, 2017, shown are overall and seasonal trends.** The dashed, red line indicates the beginning of the transition year.

For the 5–49 years old, we found no significant changes in trend and level of the monthly incidence of PCV10 ST-IPD, non-PCV10 ST ex 6A-/19A-IPD, all serotyped IPD and overall IPD in the post-period, compared to the pre-period. The monthly average IRs of ST 3 IPD, ST 6A IPD and ST 19A IPD remained almost unchanged between the pre- and post-period (Fig 2, Tables 2 and 3).

**Table 3:** Trend change in the monthly incidence during the post-period and level change at the begin of the post-period of the intervention, replacement and net change outcomes, controlled for the baseline among the 5–49 and ≥50 years old, and additionally among the age-subgroups 50–59 and ≥60 years, Austria, January 2009-February 2017.

			Trend change	Level change
Age-group	Outcome category	IPD by ST	% change/month	% change (95% CI)
5–49	intervention	PCV10 ST-IPD	+1.0 (−1.8; +3.9)	+22.9 (−48.5; +207.6)
	replacement	non-PCV10 ex ST 6A-/19A-IPD	−0.7 (−3.7; +2.2)	−1.0 (−57.6; +157.3)
	net change I	serotyped IPD	+0.7 (−1.1; +2.6)	+11.5 (−37.2; +100.3)
	net change II	overall IPD	+1.0 (−0.7; +2.8)	+10.0 (−35.7; +90.2)
≥50	intervention	PCV10 ST-IPD	−1.7 (−3.1; −0.2)	−48.2 (−67.1; −18.2)
	replacement	non-PCV10 ex ST 6A-/19A-IPD	−0.9 (−2.2; +0.5)	−27.6 (−51.9; +11.0)
	replacement	3 IPD	+0.2 (−2.0; +2.5)	−21.5 (−59.8; +60.4)
	net change I	serotyped IPD	−0.9 (−2.1; +0.3)	−35.9 (−55.2; −8.3)
	net change II	overall IPD	−1.0 (−2.1; +0.2)	−32.8 (−53.1; −3.9)
50–59	intervention	PCV10 ST-IPD	+0.3 (−3.1; +3.8)	−54.9 (−84.6; +33.9)
	replacement	non-PCV10 ex ST 6A-/19A-IPD	−1.4 (−3.8; +1.1)	−46.7 (−74.2; +12.3)
	net change I	serotyped IPD	−0.6 (−2.5; +1.3)	−45.4 (−69.0; −2.6)
	net change II	overall IPD	−0.9 (−2.7; +0.9)	−44.5 (−67.8; −3.6)
≥60	intervention	PCV10 ST-IPD	−2.2 (−3.9; −0.5)	−46.9 (−68.3; −10.7)
	replacement	non-PCV10 ex ST 6A-/19A-IPD	−0.7 (−2.3; +0.9)	−20.7 (−51.0; +29.0)
	net change I	serotyped IPD	−1.0 (−2.2; +0.3)	−33.4 (−54.7; −2.0)
	net change II	overall IPD	−1.0 (−2.2; +0.3)	−29.6 (−52.5; +4.2)

### Effects of PCV10 in ≥50 years old

#### Effect on PCV10 ST-IPD

**Fig 3:**
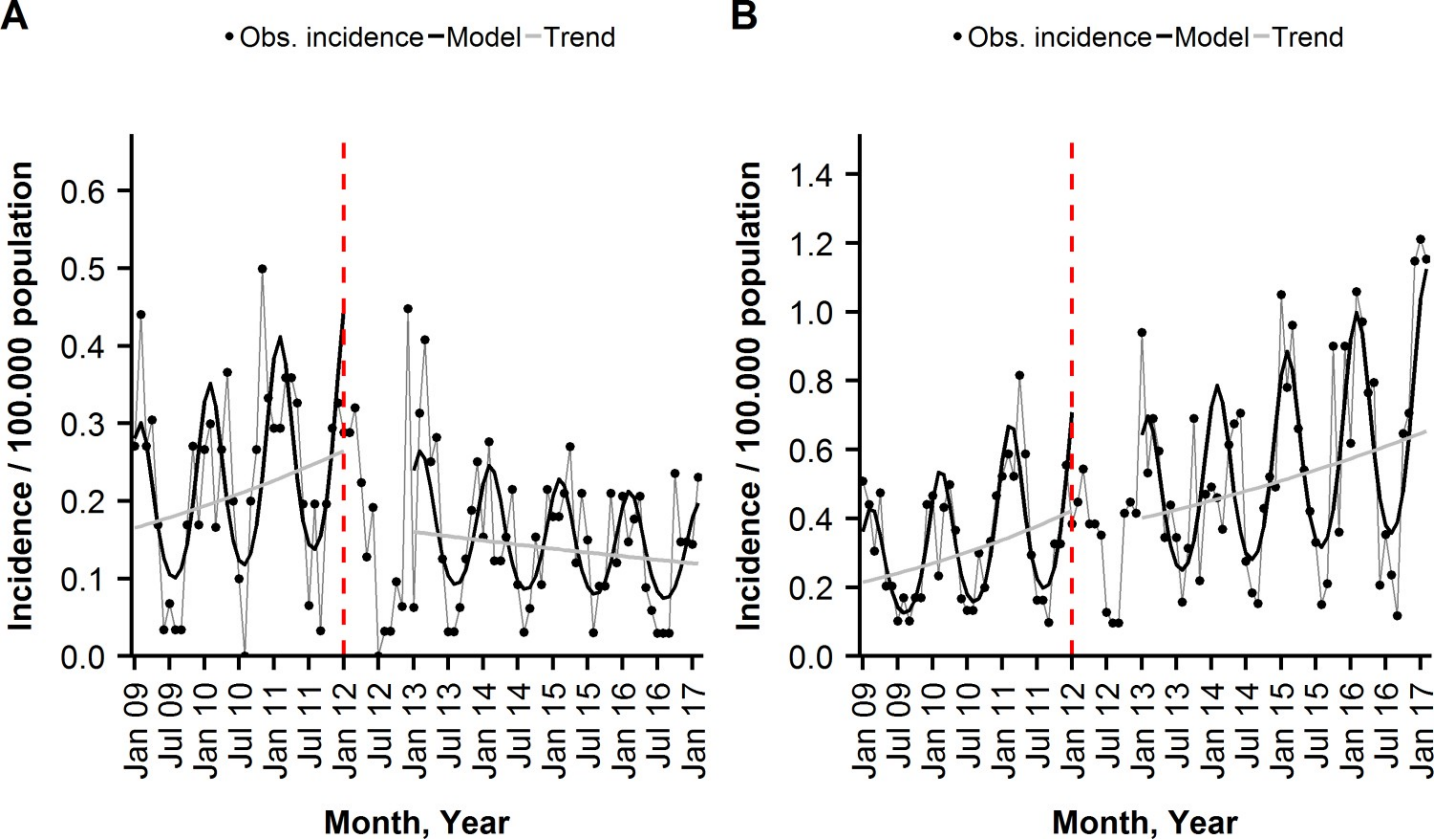
**Monthly incidence of (A) PCV10 ST-IPD and (B) non-PCV10 ex ST 6A-/19A-IPD, among the ≥50 years old, observed and modelled by a segmented negative binominal regression, Austria, January 2009-February 2017, shown are overall and seasonal trends.** The dashed, red line indicates the beginning of the transition year.

Among the ≥50 years old, the monthly average IR of PCV10 ST-IPD decreased significantly from 0.22 (pre) to 0.14 (post) per 100,000 pm, as shown in Table 2. The segmented regression model revealed a PCV10 ST-IPD incidence trend change of −1.7% per month (95% CI: −3.1%; −0.2%) in the post-period and incidence level drop at the beginning of the post-period of −48.2% (95% CI: −18.2%; −67.1%), compared to the pre-period trend and level (Fig 3, Table 3). The resulting VE was 67% (95% CI: 32%; 84%), corresponding to 462 (95% CI: 109; 1182) cases of PCV10 ST-IPD prevented (S1 Table). For the age-group ≥60 years, the pre-post change in the monthly average rate of PCV10 ST-IPD was more pronounced (Table 2). A significant incidence trend and level decrease, compared with the baseline, resulted in a VE of 71% (95% CI: 36%; 88%). Among the 50–59 years old, the trend and level changes in the PCV10-ST IPD incidence were not significant (Table 3, S1 Table).

#### Effect on PCV10-related ST-IPDs

Among the ≥50 years old, we found a significant pre-post decrease in the monthly average IR of 6A IPD (Table 2). The pre-period monthly APC was −2.9% (95% CI: −6.8%; +1.1%) and the post-period monthly APC was −1.0% (95% CI: −3.6%; +1.6%). For ST 19A IPD, there was a significant pre-post increase in the monthly average IR (Table 2). However, the monthly APC in ST 19A IPD incidence in the pre-period was +4.0% (95% CI: +0.7%; +7.7%, incidence level: 0.012/100,000) versus +1.2% (95% CI: −0.2%; +2.7%, incidence level: 0.019/100,000) in the post-period.

#### Effect on non-PCV10 ST-IPDs

In the age-group ≥50 years, the monthly average IR of the two replacement outcomes, non-PCV10 ex ST 6A-/19A-IPD and ST 3 IPD were significantly higher in the post-period, compared to the pre-period, as shown in Table 2. However, compared with the pre-period, there was no significant post-period trend or level change in the non-PCV10 ex ST 6A-/19A-IPD incidence, as revealed by the segmented time series regression analysis (Table 3, Fig 3 (B)). The same holds for ST 3 IPD (Table 3). Similar findings were made, when splitting the age group into the 50–59 and ≥60 years old (Table 3).

#### Net change

**Fig 4:**
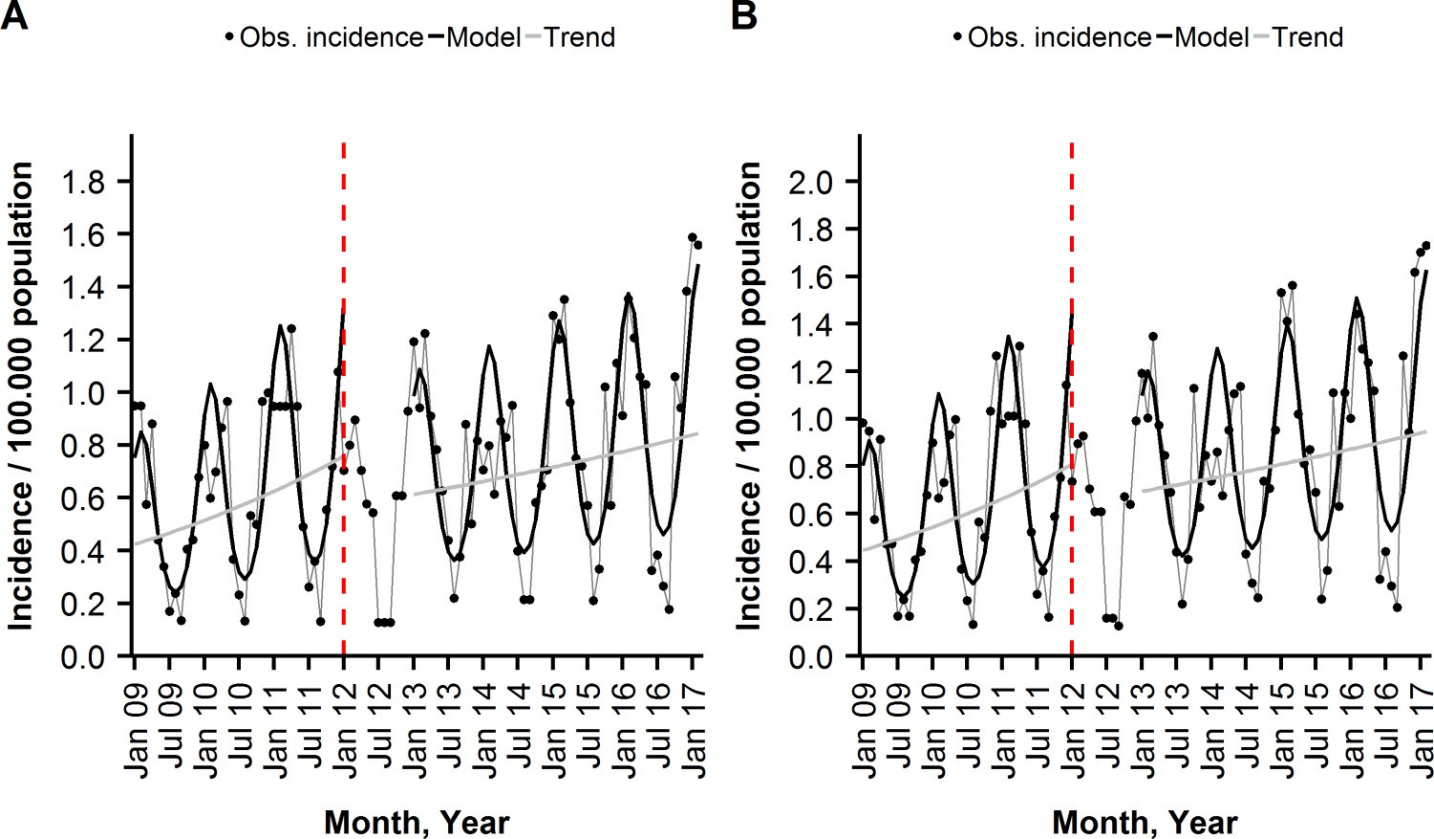
**Monthly incidence of (A) serotyped IPD, (B) overall IPD per 100,000 population among the ≥50 years old, observed and modelled by a segmented negative binominal regression, Austria, January 2009-February 2017, shown are overall and seasonal trends.** The dashed, red line indicates the beginning of the transition year.

Among the ≥50 years, old there were non-significant incidence trend changes of −0.9% and −1.0% per month in the serotyped IPD and overall IPD during the post-period, compared to the baseline trends (Table 3 and Fig 4). Together with a significant drop in the post-period incidence levels, compared to the baseline levels, it resulted in significant reductions in serotyped IPD and overall IPD of 52% (95% CI: 10%; 74%) and 51% (95% CI: 8%; 74%), respectively. After splitting the ≥50 age group, a 51% reduction in serotyped IPD (95% CI: 3%; 76%) and 49% reduction in overall IPD (95% CI: −1%; 75%) were identified for the ≥60 years. We found no vaccine net benefits in the 50–59 years old (S1 Table).

### Serotype-specific IPD among the <5 and ≥50 years old in the pre-period and early and late post-period

Among the <5 years old, ST 19A contributed the most to the IPD in in the late post-period, accounting for 8 of the 36 serotyped cases (22.2%), versus 6 of the 77 serotyped cases in the pre-period (7.8%) (Table 4, S2 Table, S1 Fig). The non-vaccine ST 3 was the second most prevalent ST in the late post-period and pre-period, accounting for 13.9% and 11.7% of the serotyped cases, respectively. The NVTs 15B and 23A, and the VTs 14, 23F, 7F appeared with two cases each in the late post-period (ranks R3-R7). The monthly average IR of IPD due to the VT 14 dropped significantly from 1.06/10^6^ pm in the pre-period (R1) to 0.20/10^6^ pm in the late post-period (R3). The remaining IPD cases of the late post-period were due to the VTs 1, 4, 6B, 19F and the NVTs 10A, 22F, 23B, 24B, 24F, 28A, 33A, 35F, 38 with one case each (IR: 0.10/10^6^ pm). The dominant occurrence of the two non-vaccine serotypes 19A and 3 in the early and late post-period did not set-off the post-period drop of the PCV10 ST-IPDs due to the STs 1, 6B, 14, 18C and 19F (Table 4, S2 Table, S1 Fig). S2 Table illustrates findings of pre-post serotype-specific IPD changes by means of early post- and late post-period IR ratio, compared with the pre-period IR per 10^6^ pm of the top 10 serotypes among the <5 years old and ≥50 years old. S1 and S2 Figs, scatterplots of the top 25 serotypes, illustrate the rank shift between pre-period and late post-period based on incidence rate per 10^6^ pm and alphanumerical order for these two age groups.

**Table 4:** Number of cases of overall IPD (N) and serotyped IPD (n_ST_), the proportion of serotyped IPD among overall IPD, the proportion of non-PCV10-IPD (n_non-PCV10_) among n_ST_, the IR of overall IPD and serotyped IPD/10^6^ pm, the serotype-specific IPD ranked from R1-R10 by IR/10^6^ pm and alphanumerically in the pre-period (2009–2011), early post-period (2013–2014) and late post-period (2015–2016) among the <5 and ≥50 years old, Austria.

		Overall IPD		Serotyped IPD		Portion of non-PCV10 IPD	Serotype-specific IPD									
							R1	R2	R3	R4	R5	R6	R7	R8	R9	R10
Age	Study period	N	IR /10^6^ pm	n_ST_	IR /10^6^	n_non-PCV10_/n_ST_ in %	IR / 10^6^ pm									
**<5**	**Pre**						**14**	**3**	**19A**	**18C**	**1**	**7F**	**11A**	**19F**	**6A**	**6B**
		108	7.63	77	5.44	46.8	1.06	0.64	0.42	0.35	0.28	0.28	0.21	0.21	0.21	0.21
	**Early post**						**14**	**19A**	**1**	**10A**	**15A**	**19F**	**24F**	**3**	**6A**	**7F**
		45	4.66	32	3.31	53.1	0.62	0.31	0.21	0.21	0.21	0.21	0.21	0.21	0.21	0.21
	**Late post**						**19A**	**3**	**14**	**15B**	**23A**	**23F**	**7F**	**1**	**10A**	**19F**
		50	4.95	36	3.56	72.2	0.79	0.49	0.20	0.20	0.20	0.20	0.20	0.10	0.10	0.10
**≥50**	**Pre**						**3**	**14**	**7F**	**19A**	**19F**	**4**	**6A**	**22F**	**9N**	**9V**
		677	6.14	638	5.79	63.2	1.08	0.48	0.42	0.34	0.27	0.26	0.26	0.24	0.21	0.18
	**Early post**						**3**	**19A**	**7F**	**14**	**4**	**6C**	**9N**	**22F**	**11A**	**23B**
		530	6.70	472	5.97	76.5	1.4	0.39	0.32	0.27	0.24	0.24	0.24	0.22	0.18	0.18
	**Late post**						**3**	**19A**	**22F**	**8**	**14**	**9N**	**23B**	**6C**	**4**	**11A**
		679	8.24	608	7.38	83.2	2.12	0.55	0.45	0.32	0.29	0.25	0.22	0.22	0.19	0.18

**Fig 5:**
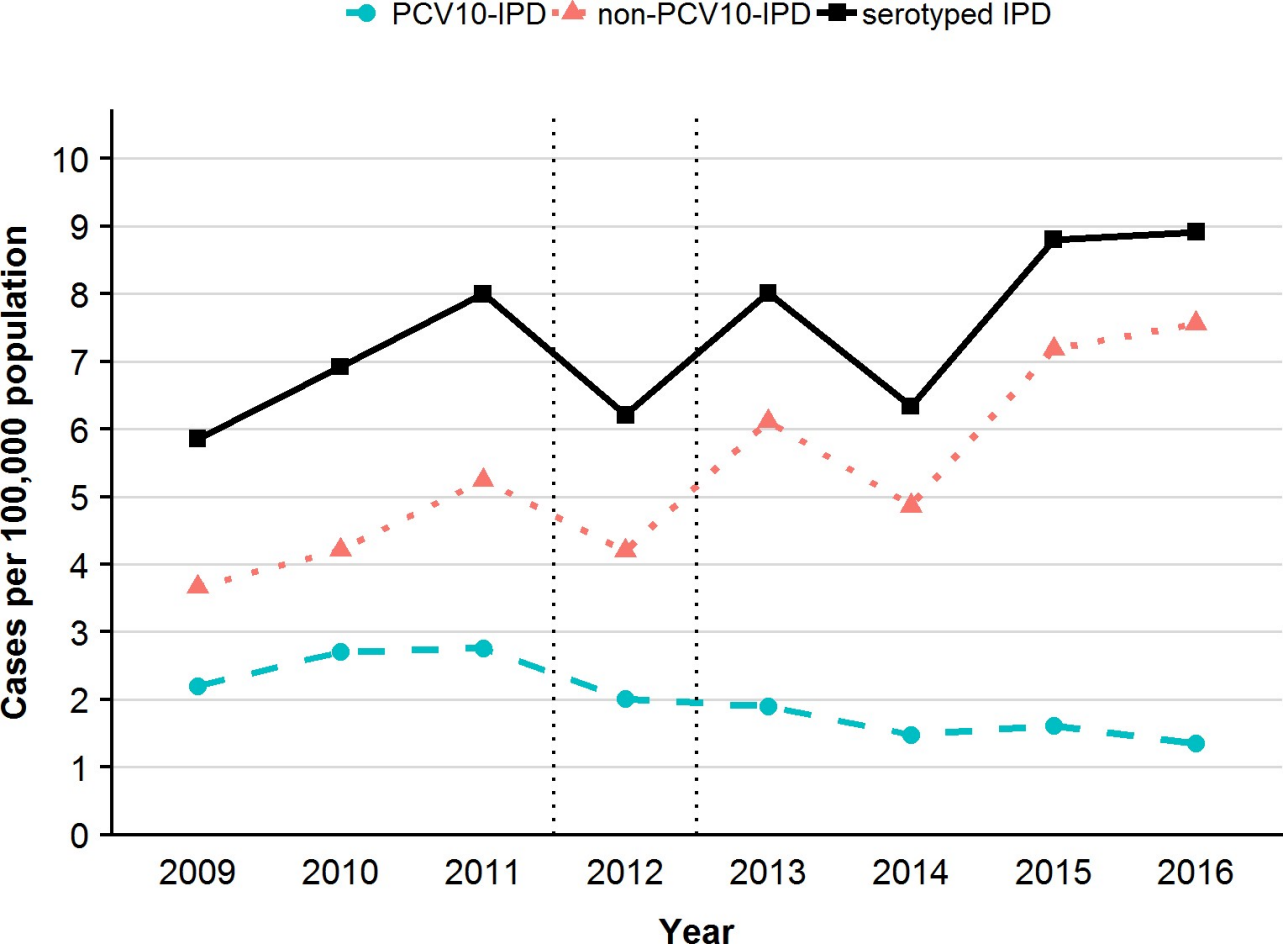
Annual incidence of serotyped IPD, and of non-PCV10 IPD and PCV10-IPD per 100,000 persons among the ≥50 years old, 2009 to 2016, Austria.

Among the ≥50 years old, the non-VTs (NVTs) constituted 83.2% of all serotyped IPD cases in the late post-period versus 63.2% in the pre-period (Table 4). The NVT 3 was the most prominent ST in the late post-period (28.7% of the serotyped IPD), as well as in the early post-period (23.5%) and pre-period (18.7%), and experienced a significant IR increase in the late post-period, compared with the pre-period (S1 Table, S2 Fig). The vaccine-related ST 19A shifted from rank 4 in the pre-period (5.9% of serotyped IPD) to rank 2 in the late post-period (accounting for 7.4% of serotyped IPD) with a significant IR increase in the late post-period (S2 Table, S2 Fig). The NVT 22F shifted from rank 8 in the pre-period (4% of serotyped IPD) to rank 3 in the late post-period (6.1% of serotyped IPD) (Table 4, S1 Table, S2 Fig) with a monthly APC of +7.2% (95% CI: 2.8%; 12.4%) in the pre-period and +2.2% (95% CI: 0.5%; 4.1%) in the post-period. NVT 8 took rank 4 in the late post-period and the NVTs 23B and 6C, the ranks 7 and 8. None of these three serotypes ranked among the top 10 STs in the pre-period. All three NVT IPDs showed a significant IR increase in the late post-period, compared to the pre-period (S2 Fig). The ST 8 IPD incidence increased by 4.7% per month (APC: 95% CI: 2.2%; 7.4%) during the post-period versus an APC of −2.6% per month in the pre-period (APC: 95% CI: −9.0%; 3.9%). In contrast, the ST 6C IPD incidence showed already a monthly increase of 13.6% (95% CI: 0.3%; 41.5%) and ST 23B IPD of 4.5% (95% CI: −3.1%; +14.0%) in the pre-period. Both were at high post-period incidence levels without post-period monthly incidence changes (ST 6C IPD APC: −0.3%/month, 95%CI: −2.6%; 2.0%; ST 23B IPD APC: + 0.2%/month, 95%CI: −2.6%; 2.0%). The monthly average IR of IPD due to VT 14 dropped significantly between the pre-period, taking rank 2 (R2), and the late post-period (R5), as also seen for the IPDs due to VT 7F (pre-period: R3; late post-period: no longer among the top 10 STs) and 19F with a shift to lower ranks (pre-period: R5; late post-period: no longer among the top 10 STs) (S2 Table, S2 Fig). The hereby-resulting overall trends of the annual incidence of serotyped IPD (PCV10 ST-IPD and non-PCV10 ST-IPD), and separated, of non-PCV10 IPD and PCV10-IPD among the ≥50 years old from 2009 to 2016 are illustrated in Fig 5.

## Discussion

We report on the first population-based study that evaluated direct and indirect effects of PCV10 as part of the publically funded Austrian childhood immunization program on IPD among the predominantly PCV7-naïve Austrian population. We analyzed the vaccine effectiveness, net vaccine benefits, and serotype-specific changes including analyses of PCV10 cross-protection and serotype-replacement.

Vaccine effectiveness in <5 years old, and in <2 years old: Among the <5 years old, the PCV10 IPD rate experienced a reduction of 58% in the post-period relative to pre-period, which corresponds to estimated 55 cases of PCV10 ST-IPD prevented by the PCV10-VP. The 2–4 years old already showed a decreasing trend in PCV10 (VT)-IPD during the pre-period, which along with slowly increasing PCV10 coverage among the <5 years old during the post-period explain the lower vaccine effectiveness among the <5 years old, compared to other observations. Finland, as a PCV7-naïve European country, is well comparable with Austria concerning the history of PCV exposure and the PCV10 2+1 doses schedule. They found in a population-based cohort-study a 92% reduction in the VT-IPD rate among the vaccine-eligible children for which a PCV10-uptake was estimated at 95%, and a 56% reduction among the 2–4 years old children [36]. In Austria, the vaccine coverage for at least two PCV10 doses among infants within the 2^nd^ year of life reached about 70% following program introduction, which suggests a larger protective effect of the PCV10-VP among older children for the following years. Among the <2 years old the PCV10-IPD rate was reduced by 77% in the post-period.

Serotype-specific IPD changes: Among the vaccine-types causing IPD, in the <5 years old, the VT 14 experienced the largest decrease in the post-period, relative to the pre-period, followed, by the vaccine-serotypes 1, 6B, 7F, 18C and 19F, showing substantial decline. Consistent with our findings, a population-based matched case-control study on PCV10 effectiveness on IPD in Brazilian children found high effectiveness against the two most common vaccine STs, the VTs 14 and 6B [48].

Cross-protection and ST-replacement: The relevant increase in the monthly ST 19A IPD incidence during the post-period, compared to the pre-period, could be attributable to VP introduction in terms of a vaccine-induced replacement. Also increasing antibiotic selection pressure, as discussed previously, could play an additional role [24]. Our data do not support cross-protection against serotype 19A among children <5 years old, which is in accordance with findings from the PCV10 using countries, The Netherlands and Sweden [49,50]. In contrast, the Finnish population based cohort-study in children, and both population-based case-control studies in Brazil and Quebec, Canada, found significant reductions in ST 19A IPDs among vaccine-eligible children following PCV10 introduction [36,48,51–53]. However, the impact in Finland was no longer significant when the follow up analysis was adjusted for a pre-VP existing decrease in the ST 19A disease incidence [27,54]. PCV10 has shown mixed evidence of direct short-lived cross-protection and little to no effect on 19A NP carriage, resulting in continued transmission and disease [27,55]. Given the increasing trend in ST 19A IPD in children <5 years old in Austria combined with lacking conclusive evidence of PCV10-evoked cross-protection against 19A, a discussion on the benefit of introducing PCV13 as part of the childhood immunization program is justified. The recent population-based before-after study by Ladhani *et al*. on IPD seven years following PCV13 introduction in England and Wales found a vaccine effectiveness of 67% for ST 19A IPD across all ages [16]. A 2017 review on the impact of PCV10 and PCV13 on ST 19A showed significantly higher functional immune responses for PCV13 than PCV10. The authors explain this by both direct impact and reductions in 19A NP carriage in children, inducing herd protection and reducing 19A IPD in non-vaccinated children and adults [56]. In the current study, the non-vaccine ST 3 was—after ST 19A - the second most prevalent ST in the <5 years old in the late post-period. Even though the descending pre-period trend and the ascending post-period trend in ST 3 IPD were non-significant, a trend reversal may indicate a ST 3 replacement in the long term among the <5 years old. [24,57].

Net Benefit: Despite the ascending post-period trend in the non-PCV10 ex ST 6A-/19A-IPD, we detected a net benefit among the <5 years old, with 33% and 35% reduction in serotyped IPD and the overall IPD. These findings are consistent with observations among children from other PCV7-naïve PCV10-countries (Finland, Kenya and Chile) and from countries using PCV7 prior to PCV10 (New Zealand and the Netherlands) [24,36,48,58].

Indirect protection of the VP in ≥50 years old: Among the ≥50 years old we detected a reduction in PCV10 ST-IPD of 67%, corresponding to 462 PCV10-IPD cases prevented due to indirect VP effect. Among the ≥60 years old, the vaccine effectiveness was even greater, at 71%. The PCV10-IPD incidence dropped at the beginning of the VP introduction, followed by a gradual decrease. This immediate incidence reduction was unexpected as there was no catch-up vaccination in place and may be explained by a preventive vaccine effect having already started in the transition year (2012) or by unknown secular trends in pneumococcal spread in the total population. It is less likely due to a direct effect of the PCV13 vaccination, because of the low vaccine uptake in this age group, as we know at least for the years 2017 and 2018. However, we did not control for a direct effect by use of the segmented regression model, because yearly PCV13 uptake rates were unavailable for 2013–2016.

As the herd protection of childhood PCV programs increases with vaccination coverage of the vaccine-eligible population and with time since VP introduction, we expect an even greater indirect protection for the coming years [15]. In The Netherlands, a PCV10–PCV7 IPD incidence reduction of 47% was observed for the 50–64 years old and of 25% for the ≥65 years old in the third year after PCV10 VP introduction [58].

Serotype-specific IPD changes, ST-replacement and cross-protection: Among the ≥50 years old, ST 3 was the most prevalent IPD causing ST in the pre- and post-period, and its average monthly incidence rate was almost two times higher in the late post-period, compared to the pre-period. However, a vaccine-attributable ST 3 replacement in IPD is rather unlikely, as the pre-existing increasing trend in the monthly incidence of ST 3 IPD continued without a significant change in the post-period. Other factors such as antibiotic selection pressure may explain the general increase in ST 3 IPD, as observed at least since 2009 in Austria [24]. ST 19A was the second most frequently observed serotype in the early and late post-period. We observed a significant increasing trend already in the pre-period, which continued to lesser extent in the post-period (meaning a smaller positive slope relative to the pre-period). However, this is unlikely due to indirect cross-protection for PCV10 against ST 19A IPD, as our data do not suggest direct cross-protection in children. This is in accordance with reports from other countries showing limited evidence, that PCV10 has any impact on NP carriage of ST 19A [27,55,59]. Also findings of studies from the PCV10-countries Finland and The Netherlands show no cross-protective herd effects in older children and adults [55,57,60,61]. Compared to the pre-period, the non-vaccine ST 8 IPD increased significantly in the adults in the post-period, which indicates a vaccine induced replacement. In summary, in the ≥50 years old, we observed a shift from the previously predominating VTs 14, 7F, 19F in the pre-period to the NVTs 3, 6C, 8, 9N, 22F, 23B in the late post-period. Our findings on the distribution of IPD causing non-PCV10 serotypes among the ≥50 years old following PCV10 introduction are consistent with findings in the PCV7- naïve PCV10-countries (Finland and Chile: concerning 3, 19A, 22F) and in countries, which used PCV7 prior to PCV10 (New Zealand: STs 3, 19A, 22F, 6C and 8; and the Netherlands: 19A, 8) [24].

Net changes: A considerable post-period drop in the levels of serotyped IPD and overall IPD together with smaller increase in the post-period, compared with pre-period level and trend resulted in a net IPD reduction for the ≥50 years old. Also The Netherlands reported an overall net IPD reduction following PCV10 introduction in older adults, compared to the PCV7 era [58]. In contrast, other PCV10-regions such as Finland, New Zealand, Quebec and PCV10-counties in Sweden reported no net IPD benefit in the older age groups due to considerable NVT-IPD increase. However, no data were presented on pre-existing trends for these countries [24,35,36,62]. As stated elsewhere, and supported by our data, secular trends in NVT-IPD can exist already prior to VP introduction.

The interrupted time series regression proved valuable in controlling for pre-intervention trends in the outcomes of interest, when we evaluated PCV10 program effects in the older populations. Due to a major quality improvement in the Austrian IPD surveillance system at the beginning of 2009, we decided to exclude previous years from our study. Hereby, we might have missed long-term secular trends or interpreted long-term fluctuations as short-term trends. However, observations from 36 months prior to VP introduction can be assumed as sufficient to provide robust estimates for trends.

To conclude, our national population-based vaccine effectiveness study brought further evidence for direct and indirect protection among children and the elderly following introduction of a childhood PCV10 VP. Adults of ≥60 years old seem to benefit the most by the herd protection. Net vaccine benefits were found in <5 and ≥50 years old. There was no evidence for PCV10-evoked cross-protection in all age groups. Our data suggest replacement possibly for ST 3 and ST 19A in <5 years old and certainly for ST 8 among ≥50 years old. Follow-up analyses of IPD surveillance data using the presented methodological approaches are strongly needed to characterize fully the magnitude of serotype replacement, to identify delayed PCV10-evoked cross-protection and to assess further vaccine-attributable IPD reduction with time after VP introduction.

## Supporting information

S1 Table Vaccine effectiveness of the childhood 2+1 PCV10 program among <5, and the age-subgroup <2 years, estimated by pre-post rate comparison, and among the ≥50 and age-subgroup ≥60 years, estimated by the segmented time series regression analysis, Austria, January, 2009-February, 2017. (DOCX)

S2 Table Serotype-specific analyses: Monthly average incidence rate of the pre-period (pre-period IR as reference), pre-early post and pre-late post IR ratios with 95% confidence interval (CI) of the top 10 serotypes of the pre-period among <5 and ≥50 years old, Austria, January, 2009-February, 2017. (DOCX)

S1 Fig Scatterplot of the top 20 IPD causing serotypes in the pre- (2009–2011) and late post-period (2015–2016) ranked by serotype-specific incidence rate (IR) /10^6^ pm and alpha-numerically among the <5 years old. Compared to the pre-period (reference rank) points above the diagonal illustrates serotypes with lower rank and points below the diagonal serotypes with higher rank in the late post-period; the size of the points correlates directly with the ratio of the serotype-specific IR between late post- and pre-period; larger points indicates higher IR ratio. Blue indicates PCV10 serotypes, red PCV13-PCV10 (i.e. 3, 6A, 19A) serotypes and black all other serotypes.
(TIFF)

S2 Fig Scatterplot of the top 25 IPD causing serotypes in the pre- (2009–2011) and late post-period (2015–2016) ranked by serotype-specific incidence rate (IR) /10^6^ pm and alpha-numerically among the ≥50 years old. Compared to the pre-period (reference rank) points above the diagonal illustrates serotypes with lower rank and points below the diagonal serotypes with higher rank in the late post-period; the size of the points correlates directly with the ratio of the serotype-specific IR between late post- and pre-period; larger points indicates higher IR ratio. Blue indicates PCV10 serotypes, red PCV13-PCV10 (i.e. 3, 6A, 19A) serotypes and black all other serotypes.
(TIFF)
