# Correction: Invasive pneumococcal diseases in children and adults before and after introduction of the 10-valent pneumococcal conjugate vaccine into the Austrian national immunization program

**DOI:** 10.1371/journal.pone.0212957

**Published:** 2019-02-21

**Authors:** Lukas Richter, Daniela Schmid, Elisabeth Eva Kanitz, Ines Zwazl, Eva Pöllabauer, Joanna Jasinska, Heinz Burgmann, Michael Kundi, Ursula Wiedermann

There are errors in S1 Table and S2 Table. Column name “VE (95%CI)” in S1 Table should be “VE in % (95%CI).” Column name “reference pre-period IR” in S2 Table should be “reference pre-period IR/10^6^.”

**S1 Table:** Vaccine effectiveness of the childhood 2+1 PCV10 program among <5, and the age-subgroup <2 years, estimated by pre-post rate comparison, and among the ≥50 and age-subgroup ≥60 years, estimated by the segmented time series regression analysis, Austria, January, 2009-February, 2017.

Age-group	Outcome category	IPD by ST	VE in % (95%CI)
<5	Intervention	PCV10 ST-IPD	58 (30; 74)
	Net change I	serotyped IPD	33 (8; 51)
	Net change II	overall IPD	35 (15; 50)
<2	Intervention	PCV10 ST-IPD	77 (53; 89)
	Net change I	serotyped IPD	53 (27;69)
	Net change II	overall IPD	48 (25; 64)
≥50	Intervention	PCV10 ST-IPD	67 (32; 84)
	Net change I	serotyped IPD	52 (10; 74)
	Net change II	overall IPD	51 (8; 74)
≥60	Intervention	PCV10 ST-IPD	71 (36; 88)
	Net change I	serotyped IPD	51 (3; 76)
	Net change II	overall IPD	49 (-1; 75)

°For the age groups 2–4, 5–49 and 50–59 years old no protective vaccine effect was detected

**S2 Table:** Serotype-specific analyses: monthly average incidence rate of the pre-period (pre-period IR as reference), pre-early post and pre-late post IR ratios with 95% confidence interval (CI) of the top 10 serotypes of the pre-period among <5 and ≥50 years old, Austria, January, 2009-February, 2017.

Age- group	pre-period rank	Serotype	Group	reference pre-period IR/10^6^	Early post-period IRR (95% CI)	Late post-period IRR (95% CI)
**<5**	1	14	VT	1.06	0.59 (0.23; 1.51)	0.19 (0.04; 0.82)
	2	3	Non-VT	0.64	0.33 (0.07; 1.51)	0.78 (0.26; 2.32)
	3	19A	Non-VT	0.42	0.73 (0.18; 2.93)	1.87 (0.65; 5.38)
	4	18C	VT	0.35	0.29 (0.03; 2.51)	-
	5	1	VT	0.28	0.73 (0.13; 4.00)	0.35 (0.04; 3.13)
	6	7F	VT	0.28	0.73 (0.13; 4.00)	0.70 (0.13; 3.83)
	7	11A	Non-VT	0.21	-	-
	8	19F	VT	0.21	0.98 (0.16; 5.84)	0.47 (0.05; 4.49)
	9	6A	Non-VT	0.21	0.98 (0.16; 5.84)	-
	10	6B	VT	0.21	0.49 (0.05; 4.69)	0.47 (0.05; 4.49)
		All serotyped		5.44	0.61 (0.40; 0.92)	0.66 (0.44; 0.97)
**≥50**	1	3	Non-VT	1.08	1.30 (1.00; 1.68)	1.97 (1.56; 2.48)
	2	14	VT	0.48	0.55 (0.33; 0.92)	0.61 (0.37; 0.98)
	3	7F	VT	0.42	0.76 (0.47; 1.23)	0.44 (0.24; 0.78)
	4	19A	Non-VT	0.34	1.17 (0.72; 1.88)	1.63 (1.05; 2.52)
	5	19F	VT	0.27	0.37 (0.17; 0.81)	0.45 (0.22; 0.91)
	6	4	VT	0.26	0.91 (0.51; 1.63)	0.74 (0.40; 1.36)
	7	6A	Non-VT	0.26	0.63 (0.32; 1.20)	0.55 (0.28; 1.09)
	8	22F	Non-VT	0.24	0.91 (0.49; 1.68)	1.90 (1.15; 3.15)
	9	9N	Non-VT	0.21	1.15 (0.63; 2.12)	1.22 (0.68; 2.21)
	10	9V	VT	0.18	0.49 (0.21; 1.15)	1.00 (0.51; 1.96)
		All serotyped		5.79	1.03 (0.92; 1.16)	1.28 (1.14; 1.43)
