# Supplementary material for: Invasive pneumococcal diseases in children and adults before and after introduction of the 10-valent pneumococcal conjugate vaccine into the Austrian national immunization program
Source: PLoS One. 2019 Jan 10;14(1):e0210081. doi: 10.1371/journal.pone.0210081 (PMC6328268; doi:10.1371/journal.pone.0210081)
Supplement: S1 Table — (DOCX) [file pone.0210081.s001.docx]

**Supporting information: S1 Table**

**S1 Table. Vaccine effectiveness of the childhood 2+1 PCV10 program among <5, and the age-subgroup <2 years, estimated by pre-post rate comparison, and among the ≥50 and age-subgroup ≥60 years, estimated by the segmented time series regression analysis, Austria, January, 2009-February, 2017.**

| **Age-group** | **Outcome category** | **IPD by ST** | **VE (95%CI)** |
| --- | --- | --- | --- |
| <5 | Intervention | PCV10 ST-IPD | 58 (30; 74) |
|  | Net change I | serotyped IPD | 33 (8; 51) |
|  | Net change II | overall IPD | 35 (15; 50) |
| <2 | Intervention | PCV10 ST-IPD | 77 (53; 89) |
|  | Net change I | serotyped IPD | 53 (27;69) |
|  | Net change II | overall IPD | 48 (25; 64) |
| ≥50 | Intervention | PCV10 ST-IPD | 67 (32; 84) |
|  | Net change I | serotyped IPD | 52 (10; 74) |
|  | Net change II | overall IPD | 51 (8; 74) |
| ≥60 | Intervention | PCV10 ST-IPD | 71 (36; 88) |
|  | Net change I | serotyped IPD | 51 (3; 76) |
|  | Net change II | overall IPD | 49 (-1; 75) |

°For the age groups 2-4, 5-49 and 50-59 years old no protective vaccine effect was detected
