# Supplementary material for: Invasive pneumococcal diseases in children and adults before and after introduction of the 10-valent pneumococcal conjugate vaccine into the Austrian national immunization program
Source: PLoS One. 2019 Jan 10;14(1):e0210081. doi: 10.1371/journal.pone.0210081 (PMC6328268; doi:10.1371/journal.pone.0210081)
Supplement: S2 Table — (DOCX) [file pone.0210081.s002.docx]

**Supporting information: S2 Table**

**S2 Table. Serotype-specific analyses: monthly average incidence rate of the pre-period (pre-period IR as reference), pre-early post and pre-late post IR ratios with 95% confidence interval (CI) of the top 10 serotypes of the pre-period among <5 and ≥50 years old, Austria, January, 2009-February, 2017.**

| **Age- group** | **pre-period rank** | **Serotype** | **Group** | **reference  pre-period IR** | **Early post-period IRR (95% CI)** | **Late post-period**  **IRR (95% CI)** |
| --- | --- | --- | --- | --- | --- | --- |
| **<5** | 1 | 14 | VT | 1.06 | 0.59 (0.23; 1.51) | 0.19 (0.04; 0.82) |
|  | 2 | 3 | Non-VT | 0.64 | 0.33 (0.07; 1.51) | 0.78 (0.26; 2.32) |
|  | 3 | 19A | Non-VT | 0.42 | 0.73 (0.18; 2.93) | 1.87 (0.65; 5.38) |
|  | 4 | 18C | VT | 0.35 | 0.29 (0.03; 2.51) | - |
|  | 5 | 1 | VT | 0.28 | 0.73 (0.13; 4.00) | 0.35 (0.04; 3.13) |
|  | 6 | 7F | VT | 0.28 | 0.73 (0.13; 4.00) | 0.70 (0.13; 3.83) |
|  | 7 | 11A | Non-VT | 0.21 | - | - |
|  | 8 | 19F | VT | 0.21 | 0.98 (0.16; 5.84) | 0.47 (0.05; 4.49) |
|  | 9 | 6A | Non-VT | 0.21 | 0.98 (0.16; 5.84) | - |
|  | 10 | 6B | VT | 0.21 | 0.49 (0.05; 4.69) | 0.47 (0.05; 4.49) |
|  |  | All serotyped |  | 5.44 | 0.61 (0.40; 0.92) | 0.66 (0.44; 0.97) |
| **≥50** | 1 | 3 | Non-VT | 1.08 | 1.30 (1.00; 1.68) | 1.97 (1.56; 2.48) |
|  | 2 | 14 | VT | 0.48 | 0.55 (0.33; 0.92) | 0.61 (0.37; 0.98) |
|  | 3 | 7F | VT | 0.42 | 0.76 (0.47; 1.23) | 0.44 (0.24; 0.78) |
|  | 4 | 19A | Non-VT | 0.34 | 1.17 (0.72; 1.88) | 1.63 (1.05; 2.52) |
|  | 5 | 19F | VT | 0.27 | 0.37 (0.17; 0.81) | 0.45 (0.22; 0.91) |
|  | 6 | 4 | VT | 0.26 | 0.91 (0.51; 1.63) | 0.74 (0.40; 1.36) |
|  | 7 | 6A | Non-VT | 0.26 | 0.63 (0.32; 1.20) | 0.55 (0.28; 1.09) |
|  | 8 | 22F | Non-VT | 0.24 | 0.91 (0.49; 1.68) | 1.90 (1.15; 3.15) |
|  | 9 | 9N | Non-VT | 0.21 | 1.15 (0.63; 2.12) | 1.22 (0.68; 2.21) |
|  | 10 | 9V | VT | 0.18 | 0.49 (0.21; 1.15) | 1.00 (0.51; 1.96) |
|  |  | All serotyped |  | 5.79 | 1.03 (0.92; 1.16) | 1.28 (1.14; 1.43) |
