# Supplementary figures and images for: Invasive pneumococcal diseases in children and adults before and after introduction of the 10-valent pneumococcal conjugate vaccine into the Austrian national immunization program
Source: PLoS One. 2019 Jan 10;14(1):e0210081. doi: 10.1371/journal.pone.0210081 (PMC6328268; doi:10.1371/journal.pone.0210081)

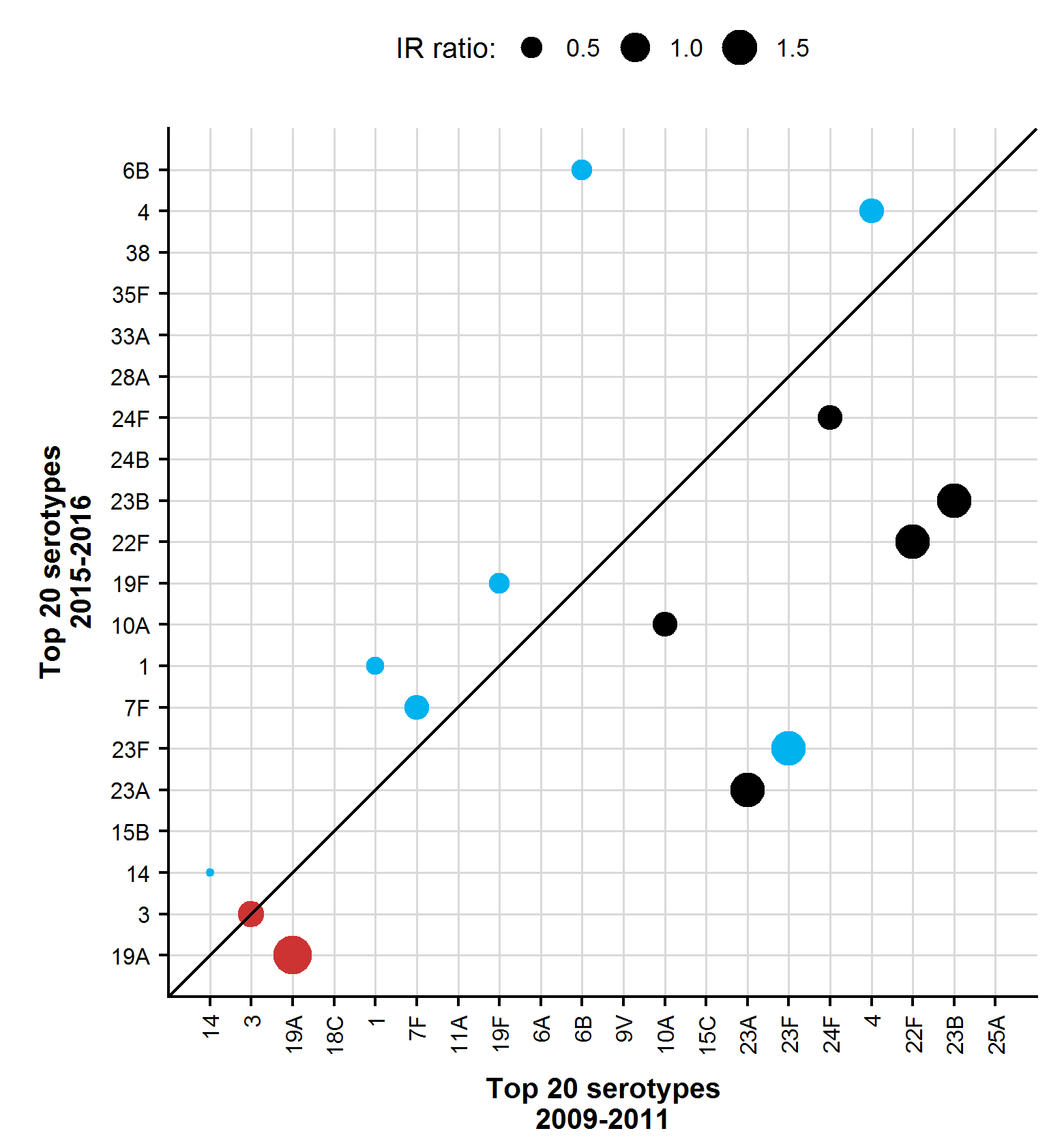

Supplement: S1 Fig — Compared to the pre-period (reference rank) points above the diagonal illustrates serotypes with lower rank and points below the diagonal serotypes with higher rank in the late post-period; the size of the points correlates directly with the ratio of the serotype-specific IR between late post- and pre-period; larger points indicates higher IR ratio. Blue indicates PCV10 serotypes, red PCV13-PCV10 (i.e. 3, 6A, 19A) serotypes and black all other serotypes. (TIFF) [file pone.0210081.s003.tiff]

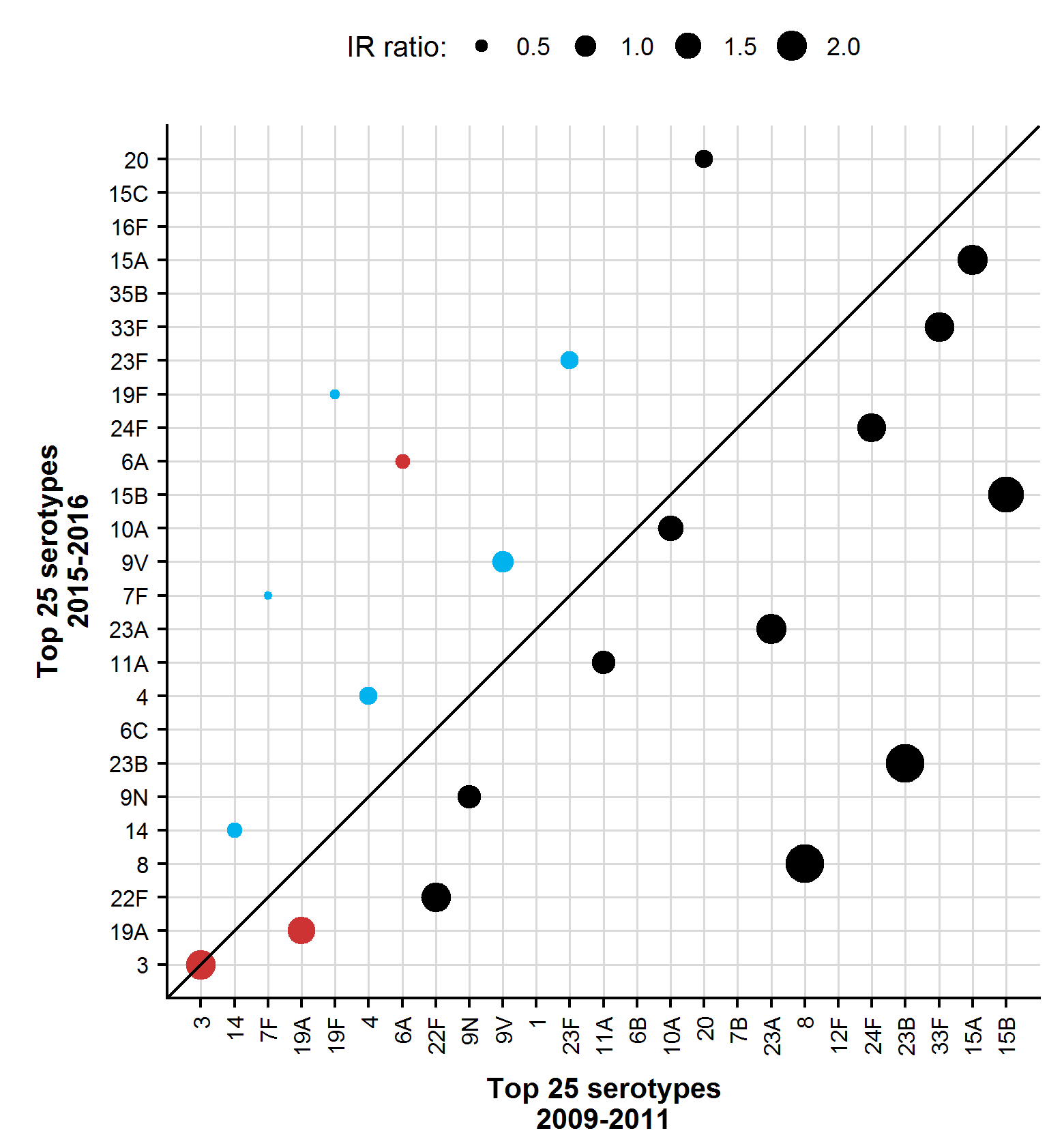

Supplement: S2 Fig — Compared to the pre-period (reference rank) points above the diagonal illustrates serotypes with lower rank and points below the diagonal serotypes with higher rank in the late post-period; the size of the points correlates directly with the ratio of the serotype-specific IR between late post- and pre-period; larger points indicates higher IR ratio. Blue indicates PCV10 serotypes, red PCV13-PCV10 (i.e. 3, 6A, 19A) serotypes and black all other serotypes. (TIFF) [file pone.0210081.s004.tiff]
